# Nucleophosmin Interacts with PIN2/TERF1-interacting Telomerase Inhibitor 1 (PinX1) and Attenuates the PinX1 Inhibition on Telomerase Activity

**DOI:** 10.1038/srep43650

**Published:** 2017-03-03

**Authors:** Derek Hang-Cheong Cheung, Sai-Tim Ho, Kwok-Fai Lau, Rui Jin, Ya-Nan Wang, Hsiang-Fu Kung, Jun-Jian Huang, Pang-Chui Shaw

**Affiliations:** 1 Centre for Protein Science and Crystallography, School of Life Sciences, The Chinese University of Hong Kong, Shatin, N.T., Hong Kong, China; 2 Laboratory of Tumor and Molecular Biology, Beijing Institute of Biotechnology, Beijing, China; 3 Stanley Ho Center for Emerging Infectious Diseases, Li Ka-Shing Medical Institute, The Chinese University of Hong Kong, Shatin, N.T., Hong Kong, China

## Abstract

Telomerase activation and telomere maintenance are critical for cellular immortalization and transformation. PIN2/TERF1-interacting telomerase inhibitor 1 (PinX1) is a telomerase regulator and the aberrant expression of PinX1 causes telomere shortening. Identifying PinX1-interacting proteins is important for understanding telomere maintenance. We found that PinX1 directly interacts with nucleophosmin (NPM), a protein that has been shown to positively correlate with telomerase activity. We further showed that PinX1 acts as a linker in the association between NPM and hTERT, the catalytic subunit of telomerase. Additionally, the recruitment of NPM by PinX1 to the telomerase complex could partially attenuate the PinX1-mediated inhibition on telomerase activity. Taken together, our data reveal a novel mechanism that regulates telomerase activation through the interaction between NPM, PinX1 and the telomerase complex.

Telomeres are G-rich non-coding tandem-repeated DNA sequences located at the ends of eukaryotic linear chromosomes that preserve the coding region and maintain chromosomal integrity. A human telomere consists of TTAGGG repeats extended in the 5′ to 3′ direction from double-stranded telomeric DNA to the single-stranded end of the telomere^1^,^2^. Telomeres are bound by the shelterin complex, which includes TRF1, TRF2, PotI, TPP1, TIN2 and RAP1^3^,^4^,^5^,^6^, serving to protect and regulate the length of telomeres. Owing to the “end replication problem” caused by DNA polymerase, telomeres shorten after each cell cycle^7^. When the telomere reaches a critical length, the chromosome ends are sensed as double-stranded breaks^8^. This process triggers the DNA damage response machinery and leads to cellular senescence or apoptosis^9^,^10^. Telomerase, which consists of hTERT and hTR components, is responsible for the maintenance of telomeres. Telomerase activity is absent in most somatic cells, and these cells are subjected to replicative senescence programmed by the cell cycle-dependent telomere shortening. By contrast, approximately 85% of immortal cancer cell lines express high level of telomerase and maintain a short telomere length^11^,^12^. Therefore, the presence of telomerase is critical for cell immortalization and tumorigenesis.

PinX1 can directly interact with hTERT and hTR^13^. This protein is a potent telomerase inhibitor that can suppress telomerase enzymatic activity and shorten the telomeres upon overexpression^14^. PinX1 expression is up-regulated in glioma tissues^15^, and it has been revealed that silencing PinX1 enhances telomerase activity and leads to telomere shortening at the same time^16^. Moreover, the anti-cancer drug anthracyclines, has been shown to down-regulate PinX1 by disrupting the association between telomeres and telomerase^17^. In a previous study, we found that the silencing of PinX1 disrupted the recruitment of telomerase to telomeres^18^. This result suggested that PinX1 is not solely a negative regulator; it may act as a mediator in telomerase recruitment or activation, likely with assistance from some of its interacting partners.

In this study, we identified nucleophosmin (NPM) as a novel PinX1 interacting partner. Nucleophosmin is a nucleolar protein involved in many cellular processes, including acting as a molecular chaperone^19^, modulating ribosome biogenesis^20^ and maintaining genomic stability^21^. Importantly, NPM has been implicated in cancer as it is overexpressed in gastric, colon, ovarian and prostate cancers^22^,^23^,^24^,^25^. Moreover, NPM has been shown to have a positive correlation with telomerase activity. Upon treatment with indomethacin, an anti-cancer chemical, telomerase activity reduces drastically in parallel with the mRNA level of NPM^26^. Additionally, telomerase activity correlates with the expression of NPM in HL-60 cells, suggesting that NPM is a positive regulator of telomerase^27^,^28^. However, the underlying mechanism of NPM’s action on telomerase remains unknown.

The present report provides evidence that NPM interacts with PinX1 directly and reveals a role of the PinX1/NPM interaction in telomerase regulation.

## Results

### Identification of NPM and confirmation of the PinX1/NPM interaction

Potential PinX1 interacting partners were found by affinity chromatography using PinX1-C (aa254–328) against the nuclear fraction of a HepG2 hepatocellular carcinoma cell lysate (data not shown). The identities of the PinX1-interacting proteins were analyzed by mass spectrometry, and NPM was found.

**Figure 1:**
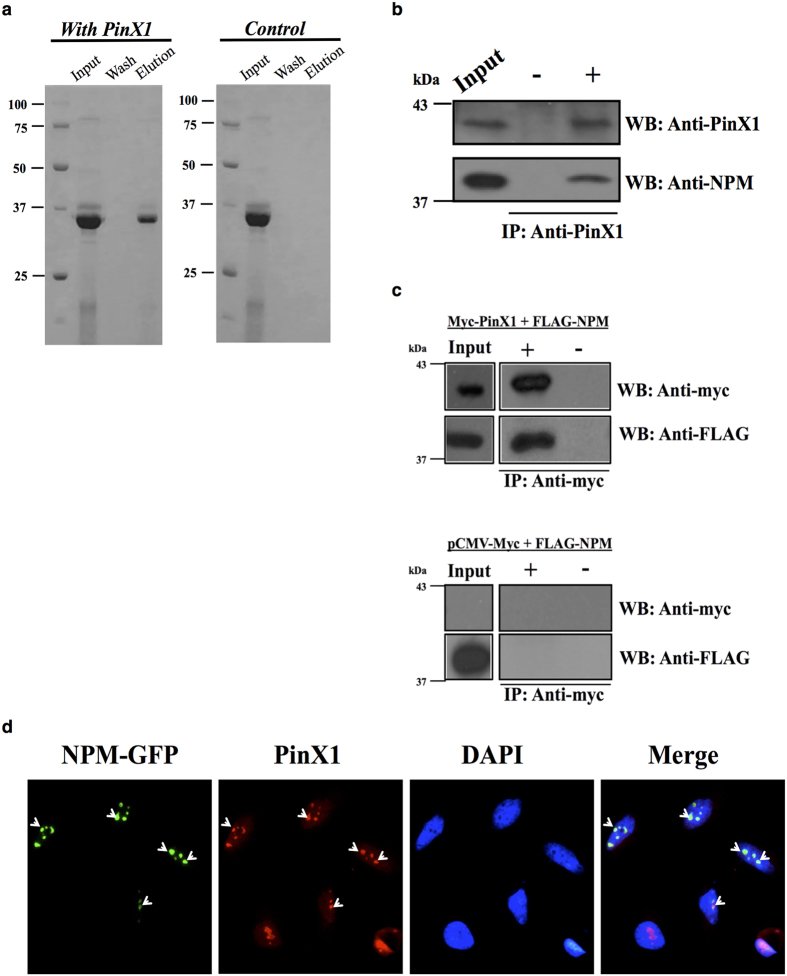
Interaction between PinX1 and nucleophosmin *in vitro* and in cells. (**a**) Direct interaction of PinX1 and NPM *in vitro*. 3 mg of purified PinX1 was immobilized on an NHS column and 1 mg of purified NPM was loaded and allowed to interact with PinX1 in the column. A control column was prepared using the same immobilization procedures but without the addition of PinX1. (**b**) Endogenous PinX1 in HEK293T cells was precipitated by anti-PinX1 and protein A beads. The ‘−’ lane shows the antibody negative control. The western blots were analyzed using anti-PinX1 (~42 kDa) and anti-NPM (~37 kDa) antibodies. (**c**) Myc-PinX1 and FLAG-NPM were co-transfected into HEK293T cells. An anti-myc antibody (9B11) and protein A beads were used to isolate the immunoprecipitates. The empty vector pCMV-myc and FLAG-NPM were co-transfected as a control for the anti-myc antibody. The western blots were analyzed using anti-myc and anti-FLAG antibodies. (**d**) Immunofluorescence showing the co-localization of PinX1 and GFP-tagged NPM in HeLa cells. Endogenous PinX1 (Red) and NPM-GFP (Green) were detected by observing the immunofluorescence in HeLa cells by fluorescence microscopy. The DAPI (Blue) panel shows nuclei staining. The merged panel shows the superimposed PinX1 and NPM images.

To verify the direct interaction between PinX1 and NPM, purified PinX1 was immobilized onto an NHS column, and purified NPM was then passed through the column. NPM was eluted from the PinX1 immobilized column but not the control column, showing that the interaction between PinX1 and NPM *in vitro* is specific (Fig. 1a). To confirm the endogenous interaction between PinX1 and NPM, co-immunoprecipitation of endogenous PinX1 and NPM was performed using HEK293T cells. NPM was co-immunoprecipitated by an anti-PinX1 antibody (Fig. 1b). This result verifies the PinX1/NPM association *in vivo*. In addition, myc-tagged PinX1 and FLAG-tagged NPM were transiently co-transfected into HEK293T cells. Consistently, FLAG-NPM was detected in the myc-PinX1 immunoprecipitate but not in the empty myc-vector control immunoprecipitate (Fig. 1c). The co-localization of endogenous PinX1 and GFP-tagged NPM was examined by immunofluorescence. GFP-tagged wild-type NPM was transfected into HeLa cells. Endogenous PinX1 was then immuno-stained using the corresponding antibody to demonstrate PinX1/NPM co-localization in the nucleus (Fig. 1d). The direct interaction between PinX1 and NPM was evident from these data.

### PinX1/NPM/hTERT associate as a complex inside the cell

**Figure 2:**
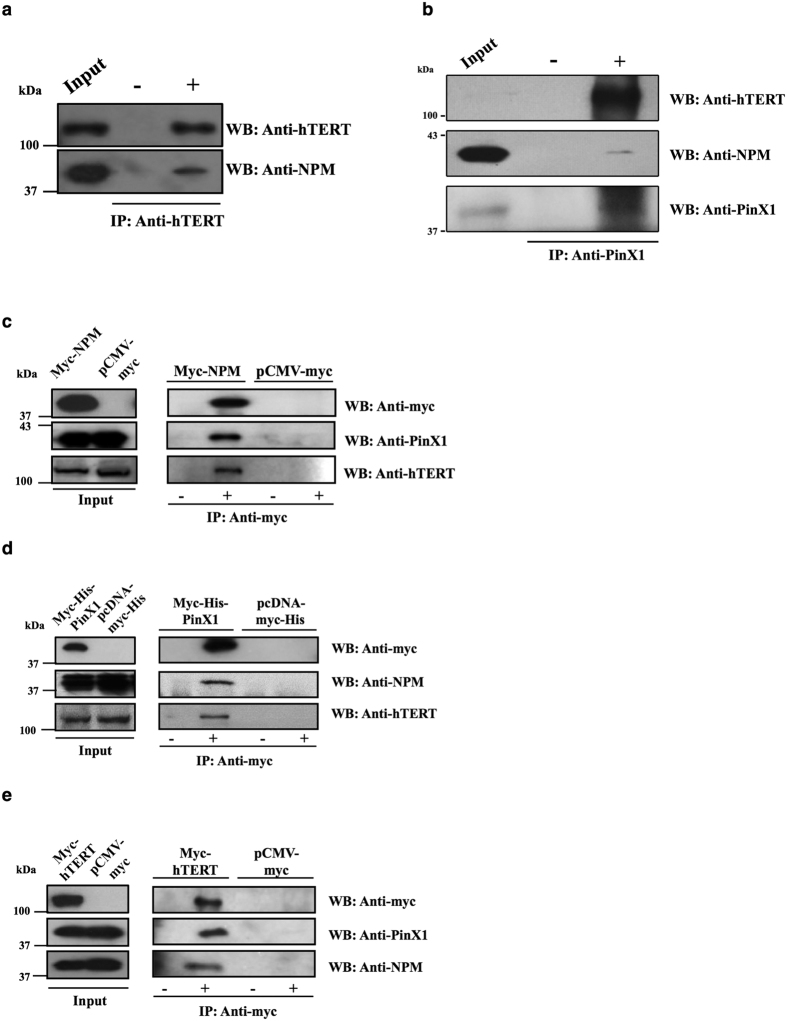
Co-immunoprecipitation showing that PinX1, NPM and hTERT are associated as a complex inside the cell. (**a**) Endogenous hTERT was immunoprecipitated by the anti-hTERT antibody from an HEK293T lysate. The presence of NPM in the precipitate was detected by an anti-NPM antibody. Antibody negative controls were included. The input controls are shown in the left panels. (**b**) Endogenous PinX1 was immunoprecipitated by an anti-PinX1 antibody from an HEK293T lysate. The presence of PinX1, hTERT and NPM in the precipitate was detected by anti-PinX1, anti-hTERT and anti-NPM antibodies. Antibody negative controls were included. The input controls are shown in the left panels. 5% of total lysates was loaded for SDS-PAGE. (**c**) Immunoprecipitation experiments were carried out in FLAG-PinX1- and pCI-neo hTERT-transfected HEK293T lysates with Myc-NPM as bait. Anti-PinX1 and anti-hTERT antibodies were used to detect the corresponding proteins. Antibody negative and empty vector controls were included in each set of experiment. The input controls are shown in the left panels. (**d**) Immunoprecipitation experiments were carried out in FLAG-NPM- and pCI-neo hTERT-transfected HEK293T lysates with Myc-His-PinX1 as bait. Anti-NPM and anti-hTERT antibodies were used to detect the corresponding proteins. Antibody negative and empty vector controls were included in each set of experiment. The input controls are shown in the left panels. (**e**) Immunoprecipitation experiments were carried out in FLAG-PinX1- and FLAG-NPM-transfected HEK293T lysates with Myc-hTERT as bait. Anti-PinX1 and anti-NPM antibodies were used to detect the corresponding proteins. Antibody negative and empty vector controls were included in each set of experiment. The input controls are shown in the left panels.

Immunoprecipitation studies were then carried out to examine whether NPM also associates with hTERT. First, the specificity of the anti-hTERT antibody was validated (Supplementary Fig. 1a). To ensure that the proteins were not non-specifically precipitated by random IgG in the assay, an immunoprecipitation experiment was performed with exogenous IgG (Supplementary Fig. 1b). In the immunoprecipitation experiment, NPM was found in the hTERT immunoprecipitate, demonstrating the NPM/hTERT association in cells (Fig. 2a). Additionally, an endogenous co-immunoprecipitation experiment was performed using an anti-PinX1 antibody. hTERT and NPM were found in the PinX1 immunoprecipitate, suggesting the association of endogenous PinX1, NPM and hTERT (Fig. 2b). To further investigate the association of NPM, PinX1, and telomerase, immunoprecipitation experiments were performed using myc-tagged NPM (Fig. 2c), myc-tagged PinX1 (Fig. 2d) and myc-tagged hTERT (Fig. 2e) as bait. It was found that PinX1, NPM and hTERT were present in the myc immunoprecipitates (Fig. 2c,d,e). Taken together, these data suggested that PinX1/NPM/hTERT associate as a complex.

### Nucleophosmin interacts with the C-terminal region of PinX1

**Figure 3:**
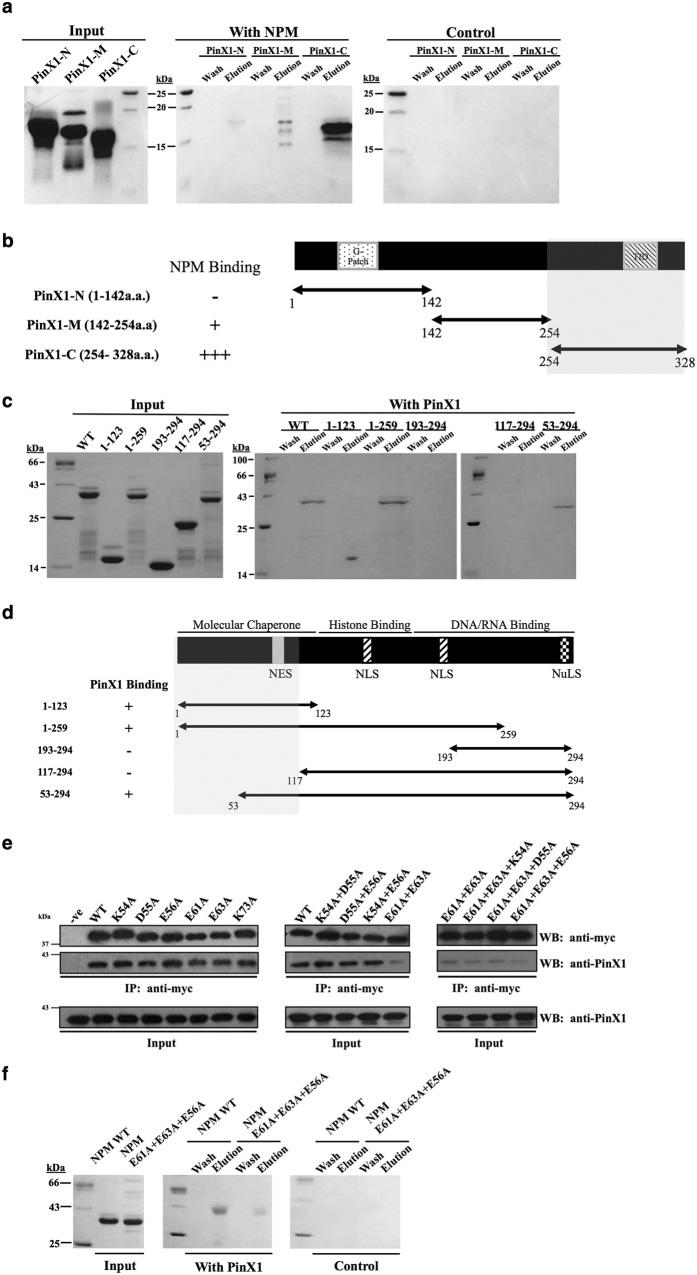
Mapping of the interaction between PinX1 and NPM. (**a**) The mapping of the NPM interaction site was carried out by an *in vitro* pull-down assay on an NHS column by using 2 mg of purified NPM as bait. A control column was prepared using the same immobilization procedures but without the addition of NPM. 1 mg of PinX1 truncations was used as ligands. (**b**) The schematic diagram shows the ability of the truncated PinX1 proteins to bind NPM, where ‘−’ represents a lack of specific binding and the number of ‘+’ is proportional to the strength of NPM binding. The shaded region indicates the NPM interacting region. (**c**) PinX1 interacts with the N-terminal region of NPM. The mapping of the PinX1 interaction site was carried out by an *in vitro* pull-down assay using a 3 mg purified PinX1-immobilized NHS column. The input purified NPM variants (1 mg) are shown on the left panel. (**d**) Map showing the ability of the truncated NPM proteins to bind PinX1, where ‘−‘ represents a lack of specific binding and ‘+’ indicates specific binding with PinX1. (**e**) Systematic charge-to-alanine mutations were made on the mapped NPM region and their binding abilities were evaluated by co-immunoprecipitation. The PinX1 binding abilities of single point mutant (left), double point mutants (middle) and triple point mutants (right) were tested. The NPM mutations E61A + E63A + E56A were shown to be critical in PinX1 binding. The negative control was performed by using the cell lysate expressing WT NPM but without adding the anti-myc antibody. (**f**) *In vitro* pull-down assay between purified PinX1 and NPM E61A + E63A + E56A. 3 mg of PinX1 was immobilized on an NHS-column, and the ability of PinX1 to pull down the wild-type NPM and the mutant NPM E61A + E63A + E56A variant was compared. 1 mg of NPM variant proteins was used as ligands. A control column was prepared with the same immobilization procedures but without the addition of PinX1.

The interaction between PinX1 and NPM was characterized by first mapping the NPM binding region on PinX1 by an *in vitro* pull-down assay. Purified NPM was immobilized onto an NHS column. Three purified PinX1 variants, PinX1-N (aa1–142), PinX1-M (aa142–254) and PinX1-C (aa254–328), were designed according to the domains found by Zhou and Lu^14^. These variants did not interact with the control column. PinX1-C showed a strong interaction with NPM, whereas PinX1-N and PinX1-M showed non-detectable and slight interactions with NPM, respectively (Fig. 3a,b).

### PinX1 interacts with the N-terminal region of nucleophosmin, and E56, E61 and E63 of nucleophosmin are critical for the interaction

Next, the PinX1 binding region on NPM was mapped. Purified PinX1 was immobilized on an NHS column and NPM variants systematically truncated at both the N- and C- terminal ends (NPM 1–123, NPM 1–259, NPM 193–294, NPM 117–294, and NPM 53–294) were passed through the column. Among these NPM truncations, NPM 1–123, NPM 1–259 and NPM 53–294 were able to interact with PinX1 (Fig. 3c), indicating that the PinX1 binding site is located within the N-terminal amino acids 53–117 of NPM (Fig. 3d).

To pinpoint the PinX1 interaction site, a systematic charge-to-alanine mutagenesis was performed within the mapped region, NPM 53–117. The NPM mutants were cloned into a myc tag-containing vector and co-transfected with FLAG-PinX1 into HEK293T cells for co-immunoprecipitation assays. The western blot analysis indicated that none of the single point mutants (K54A, D55A, E56A, E61A, E63A, K73A) showed a substantial decrease in affinity for PinX1 binding (Fig. 3e). Double point mutants with a pair of charge-to-alanine mutations (K54A + D55A, D55A + E56A, K54A + E56A, E61A + E63A) were then constructed and subjected to co-immunoprecipitation. The NPM E61A + E63A variant showed a substantial decrease in PinX1 binding (Fig. 3e). Triple point mutants were made based on the E61A + E63A mutation, and the NPM variant E61A + E63A + E56A showed a further decrease in PinX1 binding affinity (Fig. 3e). This result was further confirmed by an *in vitro* pull-down assay. Wild-type NPM or the NPM variant E61A + E63A + E56A was loaded onto a PinX1immobilized NHS column. The NPM variant E61A + E63A + E56A showed a substantial decrease in PinX1 binding affinity (Fig. 3f).

### Nucleophosmin associates with hTERT through the interaction with PinX1

**Figure 4:**
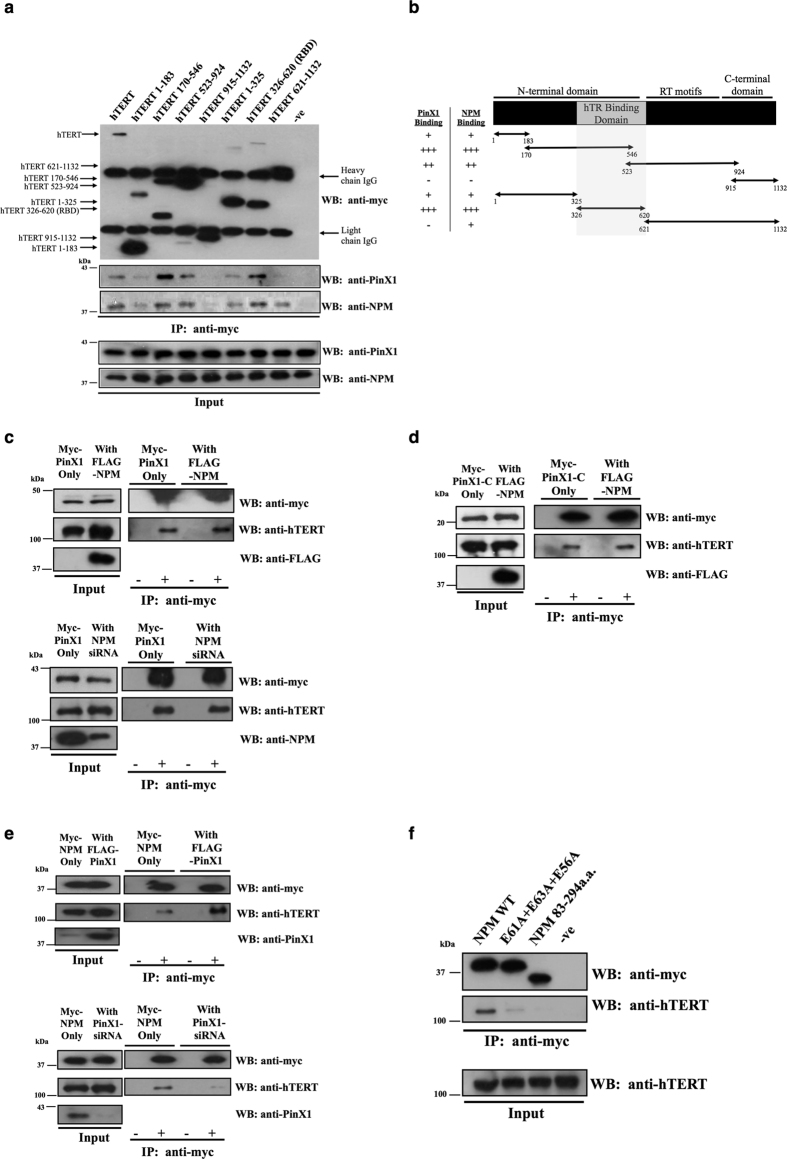
NPM does not compete with PinX1 for hTERT binding, and PinX1 acts as linker between NPM and hTERT association. (**a**) Immunoprecipitation was carried out by transfecting the myc-tagged truncations of hTERT and FLAG-PinX1 into HEK293T cells. The presence of PinX1 and endogenous NPM in the myc-precipitates was analyzed by western blotting. A negative control was performed using cell lysate expressing full-length hTERT but without adding the anti-myc antibody. (**b**) Map showing the hTERT truncations for PinX1 and NPM binding, where ‘−’ represents a lack of hTERT binding and the number of ‘+’ indicates the relative strength of PinX1 or NPM binding. The shaded region indicates the region of that interacts with both PinX1 and NPM. (**c**) The effect of NPM level on the PinX1/hTERT association was investigated in myc-PinX1- transfected lysates by western blotting. The overexpression (upper panel) and down-regulation (lower panel) of NPM were achieved by the transfection of FLAG-NPM and NPM siRNA into HEK293T, respectively. An anti-myc antibody was added to precipitate the myc-PinX1-containing complex and the relative amount of hTERT was detected by an anti-hTERT antibody. The input lysates with overexpressed or down-regulated NPM are shown in the left panel. (**d**) The effect of NPM overexpression on the binding of PinX1-C to hTERT was analyzed by western blotting. The myc-PinX1-C-containing complex was precipitated by an anti-myc antibody, and the amount of hTERT was detected by an anti-hTERT antibody. (**e**) The effect of PinX1 level on the NPM/hTERT association was investigated in myc-NPM- transfected lysates by western blotting. The overexpression (upper panel) and down-regulation (lower panel) of PinX1 were achieved by the transfection of FLAG-PinX1 or PinX1 siRNA into HEK293T cells, respectively. An anti-myc antibody was added to precipitate the myc-NPM-containing complex, and the relative amount of hTERT was detected by an anti-hTERT antibody. The input lysate with overexpressed or down-regulated PinX1 are shown in the left panel. (**f**) Immunoprecipitation of wild-type NPM, NPM E61A + E63A + E56A variant and NPM 83–294a.a. against transfected hTERT in HEK293T cells. The relative amount of hTERT was detected by an anti-hTERT antibody.

To characterize the association between hTERT, NPM, and PinX1, different hTERT variants were made according to Banik *et al*. for the study of PinX1 and NPM hTERT-binding patterns in mammalian cells, as PinX1 showed strong binding to the RNA-binding domain of hTERT in that report^13^. The hTERT variants aa1–183, aa170–546, aa523–924, aa915–1132, aa1–325, aa326–620 and aa621–1132 were cloned into a myc tag-containing vector for co-immunoprecipitation assays with NPM and PinX1 in HEK293T cells. Among the variants, aa170–546, aa523–924, and aa326–620 showed the strongest interaction with PinX1 (Fig. 4a). This result indicated that PinX1 interacts with the RNA-binding domain of hTERT, which is consistent with the previous finding^13^. Moreover, a similar relative binding strength profile was found between NPM and the hTERT variants, suggesting that NPM associates with the RNA-binding region of hTERT as well (Fig. 4a,b).

To investigate the manner of association among NPM, PinX1, and hTERT, the effect of NPM expression on the interaction between PinX1 and hTERT was studied. The overexpression or down-regulation of NPM was achieved by transient transfection of FLAG-NPM or NPM siRNA, respectively, for 48 hours. The amount of hTERT that interacted with PinX1 was slightly increased by NPM overexpression, but it was not affected by down-regulation of NPM (Fig. 4c and Supplementary Fig. 2a). Hence it is unlikely NPM competes with PinX1 for hTERT binding. Considering PinX1 possesses hTERT binding sites at both the N- and C-terminal ends and NPM only interacts with the C-terminal domain of PinX1, it is possible that NPM displaces hTERT from C-terminal domain of PinX1. To examine this possibility, the effect of NPM overexpression on the interaction between PinX1-C and hTERT was analyzed by co-immunoprecipitation. The amount of hTERT immunoprecipitated by PinX1-C did not decrease despite overexpressing NPM by the transfection of FLAG-NPM (Fig. 4d). These data suggested that NPM does not displace hTERT from PinX1 binding. Hence, the indirect association of NPM with hTERT through the PinX1 interaction is likely to account for the similar hTERT binding patterns of NPM and PinX1.

The effect of PinX1 expression on the association between NPM and hTERT was investigated to verify this speculation. The overexpression and down-regulation of PinX1 were achieved by transient transfection of FLAG-PinX1 and PinX1 siRNA, respectively. A co-immunoprecipitation analysis of NPM and hTERT was then performed. The level of hTERT immunoprecipitated by NPM was affected substantially by the PinX1 expression level. The down-regulation of PinX1 reduced the association of hTERT to NPM while overexpression of PinX1 enhanced the hTERT association to NPM (Fig. 4e and Supplementary Fig. 2b). This result indicated that PinX1 does not compete with NPM for hTERT binding, and suggested that the NPM/hTERT interaction is affected positively by the level of PinX1. Hence, we speculate that PinX1 acts as a mediator in the NPM/hTERT association and facilitates NPM loading to hTERT.

To study this possibility, the association between the NPM mutant with impaired PinX1 binding ability and hTERT was investigated. The NPM E61A + E63A + E56A variant showed a substantial reduction in hTERT association, and the NPM variant aa83–294, with the PinX1 binding site deleted, showed a complete loss of hTERT association (Fig. 4f and Supplementary Fig. 2c). This result suggested that the ability to interact with PinX1 is critical for NPM to load onto hTERT and that PinX1 bridges the association between NPM and hTERT. Thus, PinX1 is responsible for the recruitment of NPM to telomerase.

### PinX1 recruits NPM to telomerase, which attenuates the PinX1 inhibition on telomerase activity

**Figure 5:**
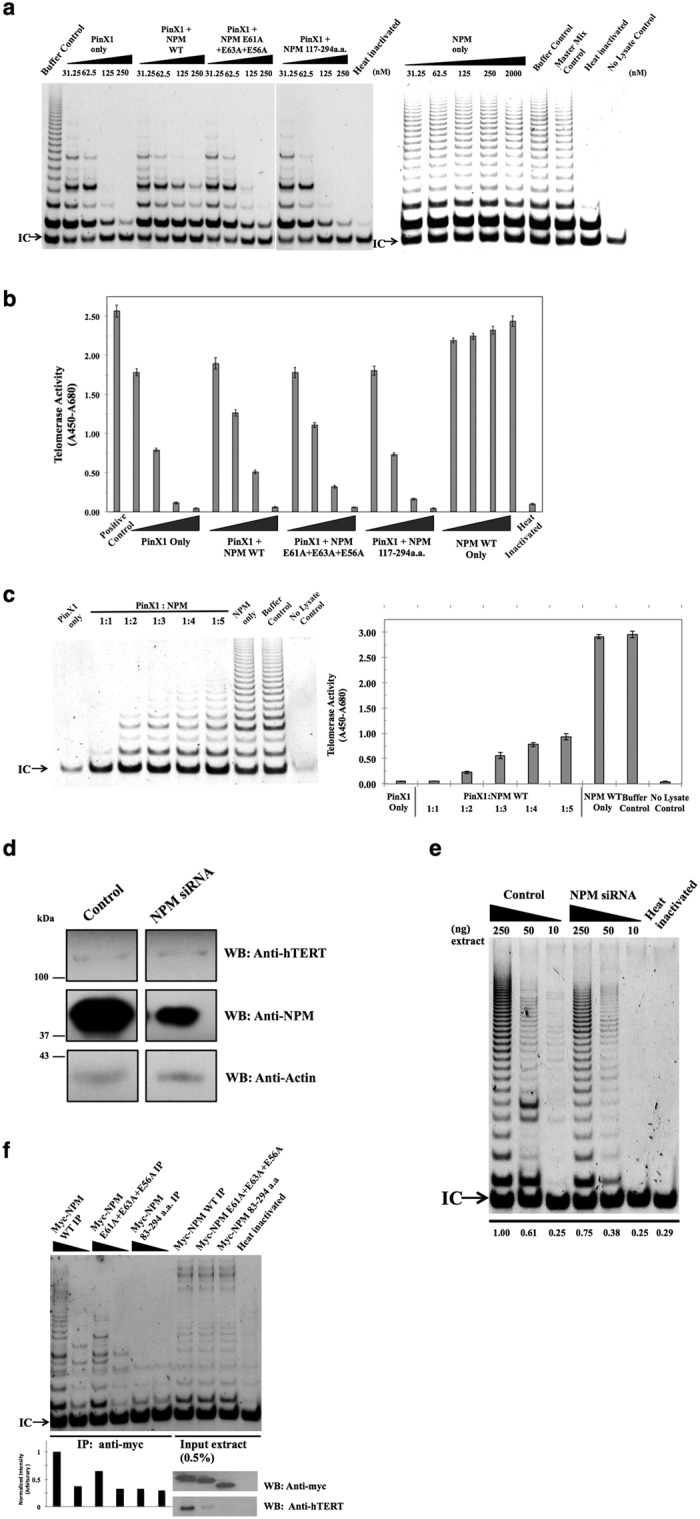
PinX1/NPM interaction is critical for NPM recruitment to active telomerase and attenuates the PinX1 inhibitory effect on telomerase activity. (**a**) The telomerase activities upon the addition of exogenous purified proteins were measured by TRAP assay. The exogenous proteins were incubated with HEK293T cell lysates on ice for 15 minutes prior to telomerase primer extension. The respective final protein concentrations in the reaction mixtures are labeled. (**b**) Telomerase activity was quantified by TRAP-ELISA as ΔA (A_450_-A_680_). The exogenous proteins were added to the reaction mixture at final concentrations of 31.25 nM, 62.5 nM, 125 nM, and 250 nM respectively prior to telomerase primer extension. (**c**) Left panel: The effect of NPM on the PinX1-mediated inhibition of telomerase activity was measured by TRAP assay. Right panel: Telomerase activity was quantified by TRAP-ELISA as ΔA (A_450_-A_680_). Increasing concentrations of wild-type NPM were incubated with a fixed amount of PinX1 (500 nM) and HEK293T lysates on ice prior to telomerase primer extension. (**d**) The effect of the down-regulation of NPM expression in HeLa cells was analyzed by western blotting. The hTERT, NPM, and actin signals are shown. (**e**) Decreased telomerase activity in NPM siRNA-transfected HeLa cells was demonstrated in a TRAP assay. The bottom panel indicates the relative band intensities in each lane against 250 ng of control extract (first lane) quantified using ImageJ software. (**f**) Telomerase activities in myc-immunoprecipitates of wild-type NPM, a mutant NPM with a disrupted PinX1 interaction site, and the NPM variant with the PinX1 binding site deleted were compared using a TRAP assay. The anti-myc and anti-hTERT input signals are shown in the lower right panel. The lower left bar chart shows the relative band intensities in each lane against the higher concentration myc-wild-type NPM immunoprecipitate (first lane) quantified using ImageJ software. The TRAP profiles shown in this figure are representative of more than three replicate experiments. IC: internal control for the PCR amplification. Buffer control: with the protein buffer. Master mix control: without purified proteins or additional buffers. Heat-inactivated control: master mix was heated at 95 °C prior to extension. No-lysate control: without the addition of cell extract.

As there was evidence to indicate that NPM associates with telomerase through a PinX1 interaction and NPM directly interacts with the C-terminal region of PinX1, which possesses the telomerase inhibitory domain, we speculated that the NPM/PinX1 interaction would affect telomerase activity. The effect of the NPM/PinX1 interaction on telomerase activity was studied by a TRAP assay. Consistent with the previous finding that PinX1 acts as a potent telomerase inhibitor, increasing amount of PinX1 added to the cell extract led to a gradual decrease in telomerase activity (Fig. 5a). Although the addition of NPM alone did not have any apparent effect on telomerase activity, it was observed that the inhibition of telomerase activity by PinX1 was partially reversed upon the addition of equal amounts of NPM together with PinX1, as indicated by the increased length of the DNA ladders in the TRAP assay and semi-quantified by the ΔA in the TRAP-ELISA assay (Fig. 5a,b). This result showed that the presence of NPM could attenuate the inhibitory effect of PinX1 on telomerase activity.

In the TRAP-ELISA assay, the recovery of PinX1 inhibition by both wild-type and variant NPM proteins was quantified. The attenuation ability of the NPM E61A + E63A + E56A mutant was weaker than that of the wild-type, whereas that of the NPM aa117–294 variant (with a complete loss of the PinX1 interaction region) was abolished, as the telomerase activity level reverted to the level of observed with PinX1 alone. At 62.5 nM, PinX1 was able to reduce the telomerase activity to 30.8% ( ±0.80%) of that of the positive control, whereas the addition of wild-type NPM and the NPM mutant along with PinX1 were able to recover the level to 49.2% ( ±1.62%) and 43.3% ( ±1.31%) of the control, respectively. At 125 nM, PinX1 reduced the telomerase activity to 4.43% ( ±0.455%) of that of the positive control. The addition of the wild-type and mutant NPM along with PinX1 was able to increase the activity to 19.9% ( ±0.887%) and 12.6% ( ±0.625%) of the positive control, respectively. The addition of the NPM aa117–294 variant to PinX1 at all concentrations did not cause a notable change to the telomerase activity compared with PinX1 alone (Fig. 5b). The attenuation ability appears to be concomitant to the ability of NPM to interact with PinX1, suggesting that NPM attenuates the PinX1-mediated inhibition of telomerase by interacting with PinX1. The attenuation effect was further demonstrated by incubating cell lysates with increasing levels of wild-type NPM in the presence of a constant amount (500 nM) of PinX1 (Fig. 5c). The telomerase activity inhibited by PinX1 could be reversed by NPM in a concentration-dependent manner. In an immunoprecipitation experiment with short-term down-regulation of the NPM level by siRNA, whereas there was no notable change in the hTERT signal (Fig. 5d) while the telomerase activity had decreased substantially in the TRAP profile (Fig. 5e).

Moreover, a substantial reduction in telomerase activity was observed in the myc-NPM E61A + E63A + E56A and myc-NPM aa83–294 variant immunoprecipitates compared with that of myc-wtNPM. The impaired ability of NPM to interact with PinX1 led to decreased hTERT association and thus reduced telomerase activity in the TRAP profile, reaffirming the importance of the association of NPM with PinX1 to the NPM/hTERT interaction (Fig. 5f). Together with the results of the immunoprecipitation experiments, our data indicate that NPM interacts with PinX1 for the association with telomerase and that this interaction results in the attenuation of the PinX1-mediated inhibitory effect on telomerase activity.

## Discussion

PinX1 is a TRF1-interacting protein that potently inhibits telomerase activity^14^. Aberrant expression of PinX1 causes telomere shortening, indicating its crucial role in telomerase regulation and telomere maintenance^16^. Meanwhile, NPM is overexpressed in many cancers, such as gastric and colon cancers^22^,^23^,^28^, and has been shown to positively correlate with telomerase activity via an unknown mechanism^26^,^27^. In this study, we have shown that NPM interacts directly with PinX1 and forms a complex with hTERT, the catalytic subunit of telomerase (Figs 1 and 2). Importantly, the recruitment of NPM by PinX1 to telomerase can attenuate the inhibitory effect of PinX1 on telomerase activity.

The expression level of NPM has been found to correlate with telomerase activity inside cells. When KATO III cancer cells were treated with indomethacin, a non-steroid anti-inflammatory drug, the telomerase activity decreased significantly in parallel with the mRNA level of NPM^26^. Moreover, higher telomerase activity was detected in an NPM overexpressing HL-60 cell extract, whereas the telomerase activity decreased HL-60 cells with down-regulated NPM expression. In addition, upon the treatment of sodium butyrate (BuONa) and vanadate, which are potent cell growth inhibitors, the level of NPM was decreased gradually and the telomerase activity was also decreased concomitantly^27^. The effect of the reduction in telomerase activity was not due to the general effect on the cell proliferation by NPM^26^. However, the underlying mechanism of how NPM positively regulates telomerase is poorly understood. Additionally, whether NPM influences telomerase activity through a protein-protein interaction or exerts its effect by altering the transcription of the telomerase regulators is unknown. Our study addressed these uncertainties and proposed a possible mechanism.

First, we found that NPM acts as a positive regulator on telomerase and can partially restore reduced telomerase activity (Fig. 5). NPM acts on the telomerase directly through a PinX1 interaction at the protein level (Fig. 5a,b). As NPM is a multi-functional protein, in the TRAP assay, purified PinX1 and NPM were added to the extract just before the assay to avoid the alteration of other genes due to the expression level of these two proteins in the cells. Our observation suggested that the alterations of telomerase activity in stable HL-60 cell lines are, at least partially, due to the protein-protein interaction between NPM and PinX1.

**Figure 6:**
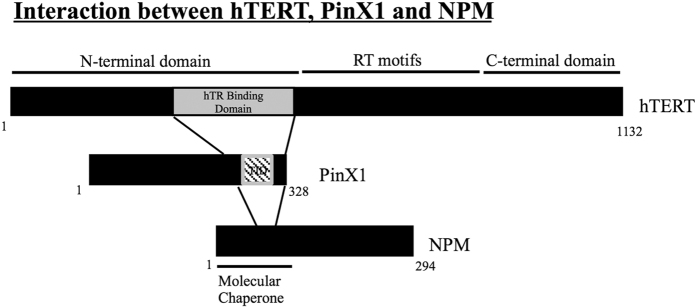
Summary of findings. Interaction between hTERT, PinX1 and NPM. The C-terminal end (including the TID domain) of PinX1 interacts with the hTR-binding domain of hTERT and links hTERT and NPM. The N-terminal domain of NPM interacts with the C-terminal domain of PinX1.

Second, our data provided evidence of the linkage between NPM and hTERT. PinX1, NPM and hTERT were found to associate together in cells, and the interaction between PinX1 and NPM is involved in telomerase activity regulation (Fig. 2). As PinX1 has been reported to directly interact with hTERT^13^, there are two possible ways that the PinX1/NPM/hTERT complex could form – hTERT may directly interact with NPM as well as PinX1, or NPM may associate with hTERT through its PinX1 interaction. We found that NPM and PinX1 both associate with the RNA binding domain of hTERT (Fig. 4), which suggested that NPM either competes with PinX1 for hTERT binding or it associates with hTERT through the PinX1 interaction. The silencing of PinX1 reduced the NPM/hTERT association, whereas the overexpression of PinX1 enhanced the association. Moreover, a reduction in hTERT association was observed for NPM variants with impaired PinX1 interaction. This result indicated that PinX1 acts as a molecular linker between NPM and telomerase for the NPM/hTERT association (Fig. 5). Through a direct interaction with the C-terminal end of PinX1, NPM can associate with the catalytic protein domain of telomerase (Fig. 6). The progression of the PinX1/NPM interaction during telomere catalysis should be further investigated.

Third, our data suggested the underlying attenuation mechanism of NPM against the PinX1-mediated inhibition on telomerase activity. NPM directly interacts with PinX1, both *in vitro* and inside cells (Fig. 1). For the latter, we are in the process of studying how these two proteins associate in different stages of cell cycle. Here, we have identified that charge-charge interactions are crucial for the PinX1/NPM association, as the NPM variant with E56, E61 and E63 mutated to alanine exhibits significant disruption in its interaction with PinX1 (Figs 3e and 4f). Although NPM interacts with the C-terminal inhibitory domain of PinX1 (Fig. 3a,b), the overexpression of NPM did not disrupt the interaction between PinX1-C and hTERT (Fig. 4c). This result suggests that NPM does not attenuate the PinX1 inhibition by displacing PinX1-C from telomerase. As PinX1 interacts with the N-terminal region of NPM (Fig. 3c,d) which is responsible for its molecular chaperone activity^19^,^29^, NPM may release the PinX1 inhibition by changing the conformation of PinX1 and thereby modulating the interaction between PinX1 and hTERT. Our finding therefore provides a direct linkage between NPM and telomerase and insight into how the overexpression or down-regulation of NPM affects telomerase activity. One of the reasons why NPM is often overexpressed in cancers could be a compensation mechanism to maintain adequate telomerase activity in these cells.

As the telomerase activity and telomere maintenance are critical for the cellular immortalization and the survival of the cancer cells^11^,^12^,^30^, the inhibition of telomerase activity has become a major target for cancer therapy. The main advantage of using telomerase as the therapeutic target is that it is expressed in major tumors of all cancer types but it is usually absent in somatic cells, thus ensuring specificity of the cancer treatment. Although NPM is a positive regulator of telomerase, targeting NPM may not be favorable due to its high abundance and multi-functionality. Instead, our data showed that the PinX1/NPM interaction has a role in telomerase activation. The disruption of the PinX1/NPM interaction may help to prevent telomerase activation and telomere maintenance and subsequently suppress the growth of certain tumor cell types. In conclusion, we have identified a novel PinX1 interacting protein, NPM, and its direct involvement in the telomerase regulatory pathway. These findings improve our understanding of the telomerase activation process, and targeting the PinX1/NPM interaction may be explored as a novel therapeutic approach for cancer treatment.

## Methods

### Cell culture and transfection

HepG2, HEK293T, HeLa (ATCC, USA) cells were cultured in Minimal Essential Medium (Gibco, Invitrogen, USA) with 10% fetal calf serum (Gibco, Invitogen, USA) in 37 °C with 5% CO_2_. Plasmid DNA was transfected into cells by Lipofectamine 2000 (Invitrogen, USA) for 24–48 hours.

### Plasmids

For the PinX1 constructs, the wild-type and variant PinX1 were cloned into pGEX-6p-2 for bacterial expression. PinX1 and PinX1-C were cloned into pcDNA3.1-mycHisA and pCMV-myc for myc-tag expression and also pCMV-TAG2B for FLAG-tag expression in mammalian cells. For the NPM constructs, the wild-type and variant NPM were cloned into pRSETA-HisSumo (from Prof. K.B. Wong, The Chinese University of Hong Kong) for bacterial expression. For mammalian cell expression, wild type NPM was cloned into pEGFP-C1, pCMV-myc and pCMV-TAG2B. The site-directed mutations were introduced by over-lapping PCR and all of the NPM mutants were cloned into pCMV-myc vector with an N-terminal myc tag. The wild-type and variant hTERT were cloned into pCMV-myc vector for expressing myc-tag protein in mammalian cells.

### Expression and purification of recombinant proteins

Constructs were expressed in *E. coli* BL21(DE3)-pLysS cells upon 0.4 mM IPTG induction. The PinX1 proteins were purified by Glutathione Sepharose (GE Healthcare, USA) and the NPM proteins were purified by HisTrap HP columns (GE Healthcare, USA) according to manufacturer’s protocol. The GST-tag and HisSumo-tags were removed by PreScission protease (GE Healthcare, USA) and Sumo protease, respectively. The tags were removed by subjecting the proteins to Superdex 75 or 200 (GE Healthcare, USA) gel filtration column with PBS as the running buffer.

### *In vitro* pull-down assay

First, 2–4 mg of purified bait protein (PinX1 or NPM) was buffer-exchanged to NHS-coupling buffer (0.2 M NaHCO_3_, 0.5 M NaCl, pH 8.3) and then immobilized on a 1 ml HiTrap NHS-activated column (GE Healthcare) according to the manufacturer’s protocol. The column was equilibrated with 5 column volumes of PBS. Then, 1–2 mg of ligand protein in 1 ml PBS was then injected into the column. The column was closed and incubated at room temperature for 30 minutes to allow the protein-protein interaction to occur. The column was then extensively washed with 20 column volumes of PBS. The bound proteins were eluted with PBS containing 1.5 M NaCl. The elution was analyzed by SDS-PAGE.

### Immunoblots and immunoprecipitation assay

Cells were washed once with PBS and 500 μl of ice-cold immunoprecipitation (IP) buffer (50 mM Tris pH 7.6, 150 mM NaCl, 1 mM EDTA, 1% Triton X-100, protease inhibitor cocktail) was added to the cells. The cells were harvested by scraping and subjected to sonication for 5 seconds. The cell lysate was centrifuged at 14000 rpm for 10 min at 4 °C. For co-immunoprecipitation assays, 200 μl of the supernatant was added to 300 μl of IP Buffer with 0.3 μg of an anti-myc antibody (9B11, Cell Signaling Technology, USA). Another 200 μl of the supernatant was added to 300 μl of IP Buffer for the antibody negative control. The tubes were incubated at 4 °C with gentle rocking for 16–18 hours. Then, 15 μl Protein A beads (50% slurry, Sigma, USA) was added to each tube and incubated at 4 °C with gentle rocking for 1.5 hours. The protein A beads were washed three times with ice-cold IP buffer, and 30 μl of 2X protein dye was added. The samples were heated at 95 °C for 10 minutes and then analyzed by western blotting. Briefly, the cell lysates and IP samples were separated by SDS-polyacrylamide gel electrophoresis and then transferred onto PVDF membranes (Millipore, USA). 12–16% of total input cell lysates was loaded unless otherwise noted. Blocking was performed with 5% skim milk in TBS-T (TBS with 0.1% Tween-20) for 30 minutes at room temperature (RT). The membranes were probed with the primary antibodies for 2 hours at RT or overnight at 4 °C followed by secondary antibodies for 2 hours at RT after washing 3 times with TBS-T for 8 minutes. The immunoblots were developed using an ECL reagent (Advansta, USA), according to the manufacturer’s protocol. The signals were developed by a film processor (Fujifilm FPM100A) and scanned with a commercial scanner. Adjustments of brightness, contrast or color balance were applied to the entire image.

The antibodies used include rabbit anti-PinX1 serum (prepared by our group), rabbit polyclonal anti-hTERT (Santa Cruz, USA), mouse monoclonal anti-myc (Cell Signaling Technology, USA), mouse monoclonal anti-FLAG (Sigma, USA), mouse monoclonal anti-β-actin (Sigma, USA), mouse monoclonal anti-NPM (Abcam, USA), goat horseradish peroxidase-conjugated anti-mouse IgG (Bio-Rad, USA), and goat horseradish peroxidase-conjugated anti-rabbit IgG (Life Technologies, USA).

For the immunoprecipitation experiments with hTERT, PinX1 or NPM down-regulation, 80 pmole of hTERT siRNA (sc-36641, Santa Cruz, USA), PinX1 siRNA or NPM siRNA (On-Target Plus SmartPool, Dharmacon, USA) was transfected using RNAiMAX (Invitrogen, USA) 5 hours prior to plasmid transfection according to the manufacturer’s protocol. The cells were harvested 48 hours post-transfection.

### Telomerase activity assays

The effect of the addition of exogenous purified proteins on telomerase activities was measured as described^14^,^31^. Purified PinX1 and NPM proteins were diluted to the desired concentrations as indicated. They were mixed and incubated for 30 minutes at room temperature to allow interaction to occur. The mixed proteins were incubated with HeLa cell extracts containing telomerase for 10 minutes on ice, and the samples were then subjected to telomere extension. The telomerase extension products were analyzed by 12.5% non-denaturing gel electrophoresis in 0.5X TBE running buffer. The gel was stained with SYBR Green I Nuclei A (Invitrogen, USA) according to the manufacturer’s instruction. For TRAP-ELISA detection, a *T*elo*TAGGG* Telomerase PCR ELISA kit (Roche, USA) was used per the manufacturer’s instructions. Negative controls were obtained by heat treatment. Telomerase activity was quantitated by a BioTek Eon reader as ΔA (A_450_-A_680_). For measuring the telomerase activity in myc immunoprecipitates, transfected cells were resuspended in Cell Lysis Buffer (10 mM Tris-HCl, pH 7.5, 20% glycerol, 50 mM KCl, 5 mM MgCl_2_, 0.01% Nonidet P-40, protease inhibitor) and incubated on ice for 30 min. An anti-myc antibody (9B11, Cell Signaling, USA) was added and incubated at 4 °C with gentle rocking for 16–18 hours. Then 20 μl of Protein A beads (50% slurry, Sigma, USA) was added to each tube and incubated at 4 °C with gentle rocking for 2 hours. The beads were washed 3 times with Cell Lysis Buffer and then 2 times with CHAPS Lysis Buffer. The Protein A beads were resuspended in 40 μl CHAPS Lysis Buffer for the TRAP assay.

### Immunofluorescence (IF)

HeLa cells were seeded and cultured on 13-mm circle cover glasses (Thermo-Menzel, Germany) in a 24-well plate. The cells were transfected accordingly (see above) if necessary. The cells were washed once with warm PBS and then fixed and permeabilized with acetone/methanol (1:1) for 10 min at −20 °C. The cells were washed twice with PBS and incubated with 2 M HCl for 20 min at room temperature. The cells were then washed with PBS once and incubated with 0.1 M boric acid for 10 min. After washing twice with PBS, the cells were blocked in Blocking Buffer (5% FBS in PBS) at room temperature for 30 min. Primary antibodies were added to the cover glass and incubated at room temperature for 2 hours. The primary antibodies used were as follows: Mouse IgM Anti-hTERT 1:3000 in 5% FBS (2C4, Abcam, USA), Mouse Anti-NPM 1:500 in 5% FBS (ab10530, Abcam, USA) and Rabbit Anti-PinX1 1:500 in 5% FBS (serum prepared by us). The primary antibodies were removed, and the cells were washed 5 times with PBS. Secondary antibodies were added to the cover glass and incubated at room temperature for 2 hours.

The secondary antibodies used were: Cy2-conjugated AffiniPure Goat Anti-mouse IgM, μ Chain Specific 1:100 in 5% FBS (Jackson ImmunoResearch, USA), Dylight 594 AffiniPure Goat Anti-mouse IgG, Fc Fragment Specific 1:200 in 5% FBS (Jackson ImmunoResearch, USA), AlexaFluor 594 Goat anti-rabbit 1:500 in 5% FBS and AlexaFluor 488 Goat anti-mouse 1:500 in 5% FBS (Invitrogen, USA). The secondary antibodies were removed, and the nuclei were stained with 0.5 μg/ml DAPI for 1 minute at room temperature. The cells were washed with PBS 3 times and then mounted on the glass slides with mounting medium (Dako, Agilent, USA).

### Microscopy

Immunofluorescence analysis was performed using a Olympus IX71 research inverted microscope with fluorescence observation. Images were acquired at 40X magnification by cooled CCD camera Olympus DP30BW. Images were merged by OLYMPUS MICRO software.

### Statistics

Data are representative of at least three replicate experiments unless otherwise noted. Values are presented in percentages or as mean ± standard deviation.

## Additional Information

**How to cite this article**: Cheung, D. H.-C. *et al*. Nucleophosmin Interacts with PIN2/TERF1-interacting Telomerase Inhibitor 1 (PinX1) and Attenuates the PinX1 Inhibition on Telomerase Activity. *Sci. Rep.* **7**, 43650; doi: 10.1038/srep43650 (2017).

**Publisher's note:** Springer Nature remains neutral with regard to jurisdictional claims in published maps and institutional affiliations.

## Supplementary Material

Supplementary Information
