# Supplementary material for: Nucleophosmin Interacts with PIN2/TERF1-interacting Telomerase Inhibitor 1 (PinX1) and Attenuates the PinX1 Inhibition on Telomerase Activity
Source: Sci Rep. 2017 Mar 3;7:43650. doi: 10.1038/srep43650 (PMC5334639; doi:10.1038/srep43650)

Supplementary Information

Nucleophosmin Interacts with PIN2/TERF1-interacting Telomerase Inhibitor 1 (PinX1) and Attenuates the PinX1 Inhibition on Telomerase Activity

Derek Hang-Cheong CHEUNG[*2], Sai-Tim HO[*2], Kwok-Fai LAU[*], Rui JIN[#], Ya-Nan WANG[#], Hsiang-Fu KUNG[†], Jun-Jian HUANG[#1], Pang-Chui SHAW*[1]

[*]Centre for Protein Science and Crystallography, School of Life Sciences and, [†]Stanley Ho Center for Emerging Infectious Diseases, Li Ka-Shing Medical Institute, The Chinese University of Hong Kong, Shatin, N.T., Hong Kong, China, and [#]Laboratory of Tumor and Molecular Biology, Beijing Institute of Biotechnology, Beijing, China

[1] To whom correspondence should be addressed: Pang-Chui Shaw, The Chinese University of Hong Kong (pcshaw@cuhk.edu.hk); Jun-Jian Huang, Beijing Institute of Biotechnology (junjianhuangbit@163.com)
[2] These authors contributed equally to this work

Supplementary Figure Legends

Supplementary Figure 1 (a) Control experiment addressing the specificity of the anti-hTERT antibody. hTERT signals in endogenous, myc-nucleoprotein-transfected (~56 kDa), and myc-hTERT-transfected (~120 kDa) HEK293T lysates. The presence of the myc-tagged overexpressed proteins was confirmed by anti-myc detection, depicted in the lower panel. A stronger signal in the myc-hTERT-transfected lysate confirmed the specificity of the anti-hTERT antibody. 5% of total lysates were loaded for SDS-PAGE. (b) Control experiment for non-specific precipitation by IgG was performed by probing myc-PinX1- and FLAG-NPM-transfected HEK293T lysates with anti-myc and anti-FLAG antibodies with 0.4 μg of mouse IgG as bait. The input controls are shown in the left panel.

Supplementary Figure 2 Full length blots of key experiments (a) Fig. 4c. The effect of NPM level on the PinX1/hTERT association was investigated in myc-PinX1- transfected lysates by western blotting. The overexpression (left panel) and down-regulation (right panel) of NPM were achieved by the transfection of FLAG-NPM and NPM siRNA into HEK293T, respectively. An anti-myc antibody was added to precipitate the myc-PinX1-containing complex and the relative amount of hTERT was detected by an anti-hTERT antibody. (b) Fig. 4e. The effect of PinX1 level on the NPM/hTERT association was investigated in myc-NPM- transfected lysates by western blotting. The overexpression (left panel) and down-regulation (right panel) of PinX1 were achieved by the transfection of FLAG-PinX1 or PinX1 siRNA into HEK293T cells, respectively. An anti-myc antibody was added to precipitate the myc-NPM-containing complex, and the relative amount of hTERT was detected by an anti-hTERT antibody. (c) Fig. 4f. Immunoprecipitation of wild-type NPM, NPM E61A+E63A+E56A variant and NPM 83-294a.a. against transfected hTERT in HEK293T cells. The relative amount of hTERT was detected by an anti-hTERT antibody. Two exposures are included.

Supplementary Figure 1

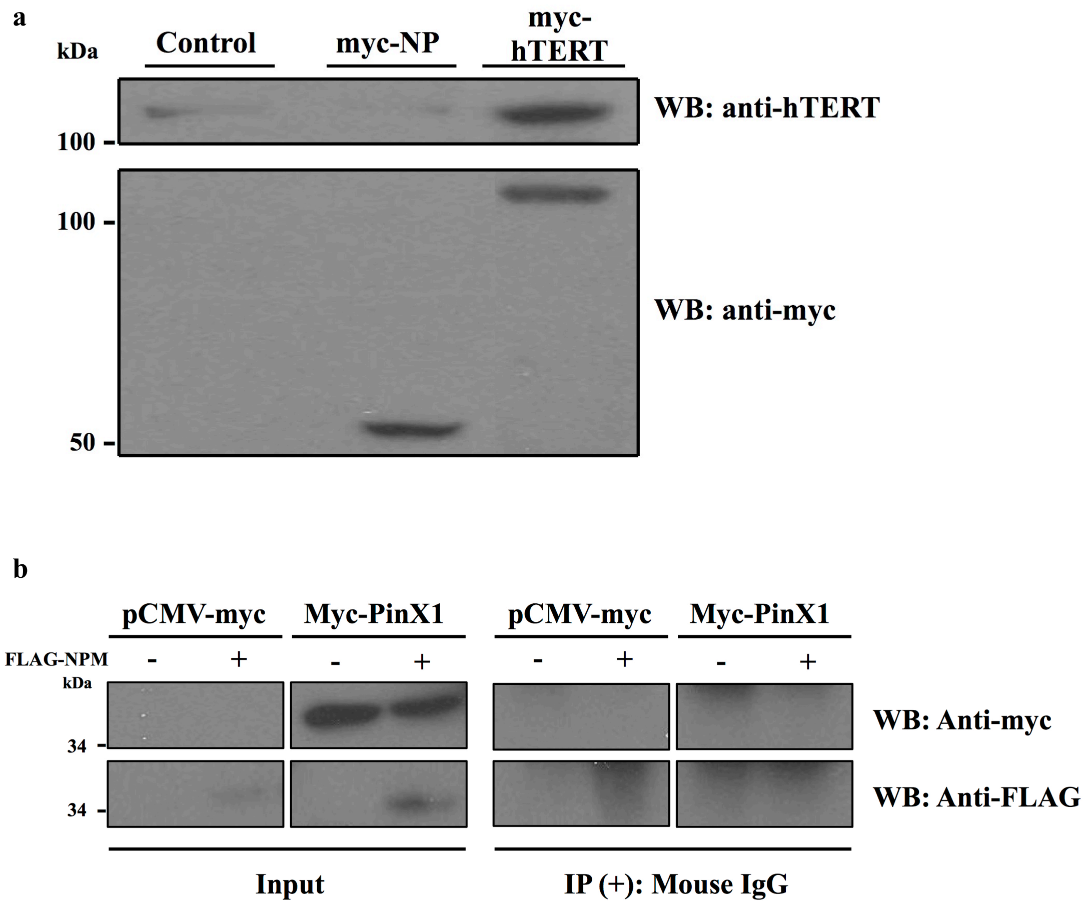

Supplementary Figure 2

a

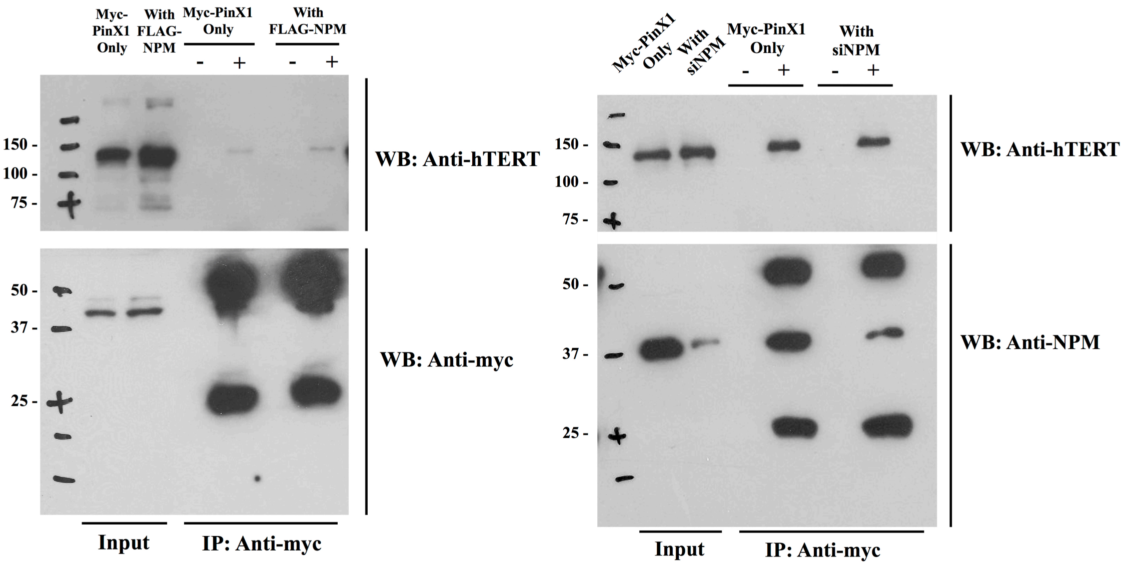

b

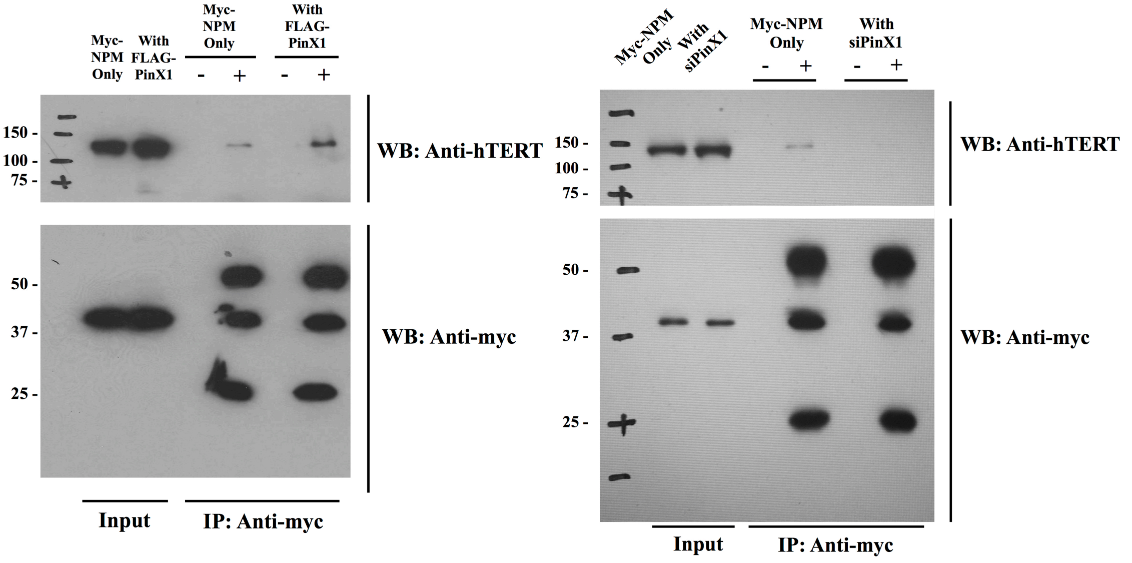

c

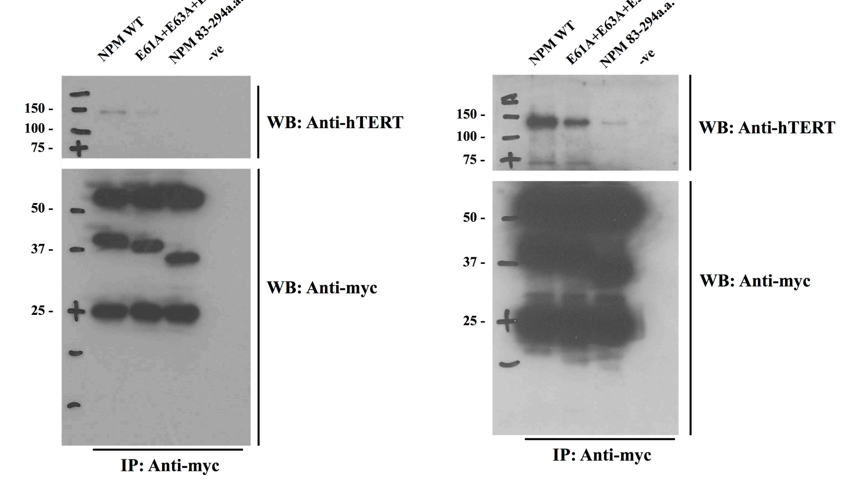

Supplement: Supplementary Information [file srep43650-s1.pdf]
